# Potential Mechanisms and Functions of Intermittent Neural Synchronization

**DOI:** 10.3389/fncom.2017.00044

**Published:** 2017-05-30

**Authors:** Sungwoo Ahn, Leonid L. Rubchinsky

**Affiliations:** ^1^Department of Mathematical Sciences, Indiana University Purdue University IndianapolisIndianapolis, IN, United States; ^2^Stark Neurosciences Research Institute, Indiana University School of MedicineIndianapolis, IN, United States

**Keywords:** neural synchrony, intermittency, neural oscillations, neural assemblies, delayed-rectifier potassium current

## Abstract

Neural synchronization is believed to play an important role in different brain functions. Synchrony in cortical and subcortical circuits is frequently variable in time and not perfect. Few long intervals of desynchronized dynamics may be functionally different from many short desynchronized intervals although the average synchrony may be the same. Recent analysis of imperfect synchrony in different neural systems reported one common feature: neural oscillations may go out of synchrony frequently, but primarily for a short time interval. This study explores potential mechanisms and functional advantages of this short desynchronizations dynamics using computational neuroscience techniques. We show that short desynchronizations are exhibited in coupled neurons if their delayed rectifier potassium current has relatively large values of the voltage-dependent activation time-constant. The delayed activation of potassium current is associated with generation of quickly-rising action potential. This “spikiness” is a very general property of neurons. This may explain why very different neural systems exhibit short desynchronization dynamics. We also show how the distribution of desynchronization durations may be independent of the synchronization strength. Finally, we show that short desynchronization dynamics requires weaker synaptic input to reach a pre-set synchrony level. Thus, this dynamics allows for efficient regulation of synchrony and may promote efficient formation of synchronous neural assemblies.

## Introduction

Synchrony of neural oscillations is believed to play important role in a variety of functions of the brain (e.g., Buzsáki and Draguhn, 2004; Colgin, 2011; Fell and Axmacher, 2011; Buzsáki and Schomburg, 2015; Fries, 2015; Harris and Gordon, 2015). Improperly organized (too excessive or too weak) synchrony is associated with several neurological and neuropsychiatric dysfunctions (e.g., Schnitzler and Gross, 2005; Uhlhaas and Singer, 2006, 2010; Oswal et al., 2013; Pittman-Polletta et al., 2015; Spellman and Gordon, 2015). However, the synchrony in cortical and subcortical circuits may not necessarily stay perfect for a prolong intervals of time (if it can be perfect at all). If synchrony is induced by transient stimulus, the transient character of this synchrony would be expected. But even in the idling dynamics of neural circuits of the brain prolonged perfect synchrony is rarely (if at all) reported.

This implies that for some intervals of time synchrony may be stronger, while for other intervals of time it may be weaker. The temporal patterns of synchrony may exhibit variations of synchrony strength yielding some average synchrony values. Few long intervals of desynchronized dynamics may be functionally different from many short desynchronized intervals, although the synchrony may be the same on the average.

Detection and quantification of the transient, varying, intermittent synchronization have been considered in the past (e.g., Hurtado et al., 2004; Le Van Quyen and Bragin, 2007). But these attempts were limited by the need in sufficiently long time-windows to obtain statistical significance, because synchronization is not an instantaneous phenomenon (Pikovsky et al., 2001). However, recent developments in time-series analysis (Ahn et al., 2011) allowed exploring the temporal patterning of synchrony on very short time-scales. The analysis of this fine temporal structure of synchronization is possible, because if some synchrony level is present on the average, then one can look at each cycle of oscillations and detect whether the signals are in the synchronous state or not.

These methods were used to study neural synchronization in several different systems: synchronization between single units and LFPs in the basal ganglia of Parkinsonian patients (Park et al., 2010; Ratnadurai-Giridharan et al., 2016), synchronization of EEG signals in healthy humans (Ahn and Rubchinsky, 2013), synchronization of LFPs between prefrontal and hippocampus circuits in normal rats and rats experiencing repetitive psychostimulant injections (Ahn et al., 2014). All these studies had one common feature: neural oscillations were observed to go out of synchrony frequently, but primarily for a short time interval. The observed synchrony level was reached by potentially very frequent, but short desynchronizations.

Since this short desynchronization dynamics was observed across different species, different conditions, and different signal types, it may be a universal feature of synchronized activity of neural systems. In this study we are providing a possible explanation for this apparent experimentally observed universality. We do so by looking for answers to two questions: what are the cellular or network mechanisms of this dynamics? What is its potential functional advantage?

We hypothesize that if this kind of dynamics is universal, it may be grounded in some general properties of neuronal excitability. In connection with this hypothesis, it is important to recall early insightful computational study (Somers and Kopell, 1993), which suggested that membrane conductances responsible for spiking help to speed up the establishment of synchrony. Here we will explore how experimentally observed short desynchronizations dynamics is defined by the kinetics of ionic channels, responsible for the generation of spikes. We also hypothesize that short desynchronization dynamics permits creation of synchronous states with weaker inputs. This may make neural systems more adaptable as they can easily create synchronous assemblies in response to synaptic or sensory inputs.

Since short desynchronization dynamics may be a generic phenomenon based on the properties of membrane channels, which are hard to alter in experiment, we use computational neuroscience techniques to study very simple conductance-based neuronal models. We alter the properties of conductances to explore their critical features for short desynchronization dynamics and investigate how coupled neurons may be efficiently entrained by external input. Models are subjected to the same kind of time-series analysis techniques as were used in earlier experimental studies. As a result, we reveal potential cellular basis of short desynchronization dynamics in the model and present its potential functional advantages.

## Methods

### Neuronal model

We use a conductance-based modified Morris-Lecar neuronal model (Izhikevich, 2007; Ermentrout and Terman, 2010). We choose it because it is a simple (perhaps, the simplest) model that directly retains membrane conductances. Even though the original Morris-Lecar model includes calcium and potassium currents, it is equivalent to a reduced classical Hodgkin-Huxley sodium-potassium model (Izhikevich, 2007; Ermentrout and Terman, 2010). So, by studying this neural model, we model neurons with sodium-potassium spiking mechanism with fast sodium and delayed rectifier potassium currents.

We consider the model in the form:

(1) $$\begin{array}{r} v\prime =\;-I_{Na}-I_{K}-I_{L}-I_{syn}+I_{app}, \end{array}$$

(2) $$\begin{array}{r} w\prime =\;\left[w_{\infty }\left(v\right)-w\right]/\tau \left(v\right). \end{array}$$

*v* is a transmembrane voltage and *w* is the gating variable of potassium current. *I*_*Na*_ = *g*_*Na*_ *m*_∞_(*v*)(*v* − *v*_*Na*_), *I*_*K*_ = *g*_*K*_*w* (*v* − *v*_*K*_) and *I*_*L*_ = *g*_*L*_(*v* − *v*_*L*_) are sodium, potassium, and leak currents; *I*_*app*_ is a constant parameter and *I*_*syn*_ is a synaptic current (see below). *g*_*Na*_, *g*_*K*_, *g*_*L*_ are the maximal conductances for the Na^+^, K^+^, and the leak currents. The functions

(3) $$\begin{array}{r} m_{\infty }\left(v\right)=\;\frac{1}{1+\exp\left(-2\left(\frac{v-v_{m_{1}}}{v_{m_{2}}}\right)\right)}, \end{array}$$

(4) $$\begin{array}{r} w_{\infty }\left(v\right)=\;\frac{1}{1+\text{exp}\left(-2\left(\frac{v-v_{w_{1}}}{\beta }\right)\right)}, \end{array}$$

(5) $$\begin{array}{r} \tau \left(v\right)=\;\frac{1}{\varepsilon }\frac{2}{\exp\left(\frac{v-v_{w_{1}}}{2\beta }\right)+\text{exp}\left(\frac{-\left(v-v_{w_{1}}\;\right)}{2\beta }\right)} \end{array}$$

are the steady-state activation functions of the gating variables of the Na^+^ and K^+^ currents, and the activation time-constant for K^+^ current. The functions *m*_∞_(*v*) and *w*_∞_(*v*) have sigmoid shapes while τ(*v*) has a unimodal shape. The term *I*_*syn*_ represents the synaptic current between cells.

We consider neurons connected with excitatory synapses adapted from Izhikevich (2007) and Ermentrout and Terman (2010). For a cell *i*, the synaptic current $I_{syn,\;i\;}=\;g_{syn}\left(v_{i}-\;v_{syn}\right)\;\sum_{j\neq i}s_{j}$, where the sum is over those cells that send synaptic inputs to a cell *i*. The synaptic variable *s* is modeled by the first-order kinetic equation in the form:

(6) $$\begin{array}{r} s\prime =\;\alpha_{s}\left(1-s\right)H_{\infty }\left(v-\theta_{v}\right)-\;\beta_{s}\;s, \end{array}$$

where $H_{\infty }\left(x\right)=1/\left[1+\exp\left(-\frac{x}{\sigma_{s}}\right)\right]$ is a sigmoidal function and *v* is the presynaptic neuron voltage (Izhikevich, 2007; Ermentrout and Terman, 2010). The parameter values are *g*_*Na*_ = 1, *v*_*Na*_ = 1, *g*_*K*_ = 3.1, *v*_*K*_ = −0.7, *g*_*L*_ = 0.5, *v*_*L*_ = −0.4, *I*_*app*_ = 0.045, *v*_*m*_1__ = −0.01, *v*_*m*_2__ = 0.15, β = 0.145, *v*_*w*_1__ = 0.08, ε = 0.02, *g*_*syn*_ = 0.005, *v*_*syn*_ = 0.5, α_*s*_ = 2, β_*s*_ = 0.2, θ_*v*_ = 0, σ_*s*_ = 0.2. We will further vary the values of ε, β, and *v*_*w*_1__ as will be described in the Results.

### Synchronization analysis

Phase analysis is frequently used to analyze synchronous neural activity of both continuous (LFP, EEG) and spiking signals (see, e.g., Lachaux et al., 1999; Hurtado et al., 2004; Le Van Quyen and Bragin, 2007). This analysis was used in experimental studies revealing prevalence of short desynchronization dynamics (Park et al., 2010; Ahn and Rubchinsky, 2013; Ahn et al., 2014; Ratnadurai-Giridharan et al., 2016). So we assume a very similar approach here. For a spiking activity, a phase of a neuron *i* is reconstructed by computing

(7) $$\begin{array}{r} \varphi_{i}\left(\text{t}\right)=\text{arctan}\left(\frac{v_{i}\left(t\right)-{\hat{v}}_{i}}{w_{i}\left(t\right)-{ŵ}_{i}}\right), \end{array}$$

where $\left({\hat{v}}_{i},\;{ŵ}_{i}\right)$ is a rest state of a neuron, set as a center of rotation in the (*v*_*i*_, *w*_*i*_)-plane. Then we consider an average synchronization index to measure the strength of the phase locking between two signals (Pikovsky et al., 2001; Hurtado et al., 2004):

(8) $$\begin{array}{r} \gamma =||\frac{1}{N}\sum_{j=1}^{N}e^{i\Delta \varphi \left(t_{j}\right)}|{|}^{2}, \end{array}$$

where Δφ(*t*_*j*_) = φ_1_(*t*_*j*_) − φ_2_(*t*_*j*_) is the phase difference, the *t*_*j*_ are the sampling points, *N* is the number of data points to be considered, and ||.|| is the absolute value of a complex number.

This phase synchronization index γ varies from 0 (lack of synchrony) to 1 (perfect synchrony). It provides an average value of phase-locking. There may be cycles of oscillations, where phase difference is close to the average value of the phase difference (phase-locked, synchronized state) and where it is not close to it (desynchronized state).

To study the fine temporal structure of the dynamics of synchronization we use the methods recently developed in Park et al. (2010) and Ahn et al. (2011). Whenever φ_1_ crossed the zero from negative to positive values, we recorded the value of φ_2_, generating a set of consecutive phase values {ϕ_*i*_}, *i* = 1, …, *N*. If the value of ϕ_*i*_ differs from the average value of ϕ_*i*_ by less than π/2 then the neurons are considered to be in a synchronized state, otherwise they are in the desynchronized state. We chose the value of the threshold to be π/2 because the experimental studies we discussed above used this value. The duration of desynchronization events is defined as the number of cycles of oscillations that the system spends in the desynchronized state minus one. The mode of the distributions of desynchronization durations is defined as the number with the highest probability of desynchronization durations.

We characterize the fine temporal structure of intermittent synchronization by quantifying the properties of distribution of desynchronization durations. We compute the relative frequencies (probabilities) of the durations of desynchronization events. This is similar to how the experimental data were characterized in the studies of the temporal patterns of synchrony (Park et al., 2010; Ahn and Rubchinsky, 2013; Ahn et al., 2014; Ratnadurai-Giridharan et al., 2016). We use the mode of the distribution of desynchronization durations and the probability to observe this mode, *p*_*mode*_. If the mode of the desynchronization duration is short, but other desynchronizations (especially longer ones) are almost as frequent, then the dynamics is not necessarily dominated by short desynchronizations. However, if *p*_*mode*_ is close to one, then all other desynchronization durations are rare.

In our approach the duration of synchronization and desynchronization intervals is measured not in the absolute time units, but in cycles of oscillations, as was done in experimental studies. It makes easier to compare synchronization patterns between rhythms of different frequency. However, as we study the differences between different desynchronization durations in the modeling, we also compare the dynamics with the same frequencies of rhythms (see Results).

## Results

**Figure 1:**
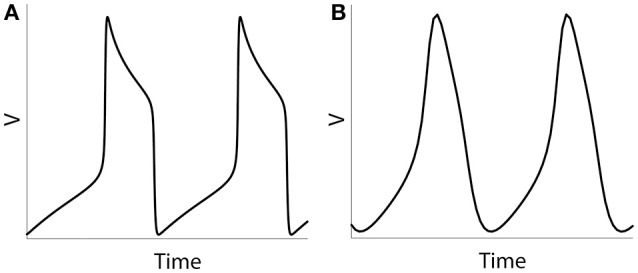
Numerical simulation of a voltage of an isolated neuron (Equations 1–5). Examples of spiking activity (ε = 0.001) **(A)** and quasi-sinusoidal activity (ε = 0.5) **(B)**.

We will study the dynamics of coupled model neurons as we vary parameters of potassium current. We do so by varying three different parameters: ε, β and *v*_*w*_1__ (see Equations 4 and 5), they all affect the effective value of activation time-constant τ(*v*) of potassium current. Larger values of τ delay activation of potassium current and promote characteristic shape of spike with very sharp rise of voltage, faster decay of voltage, and prolong interval between spikes. Lowering effective values of τ in the model (for example, by using larger values of ε) will lead a model neuron to generate less spiky and more quasi-sinusoidal profile of activity (Figure 1). By changing the values of ε, β, *v*_*w*_1__ we can study the model neurons exhibiting spiking activity like at Figure 1A as well as more sinusoidal activity like at Figure 1B (which is not necessarily very realistic, but will help in understanding the mechanisms and functions of physiological activity).

### Kinetics of voltage-gated potassium channel and the temporal patterning of synchronization

**Figure 2:**
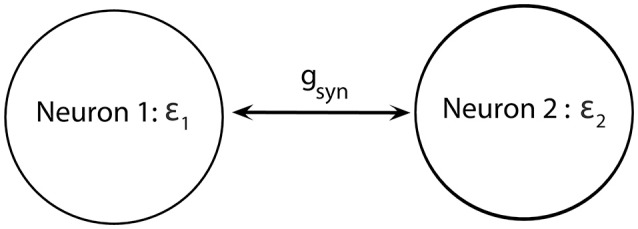
Diagram of a minimal network of excitatory coupled neurons. We use ε_1_ ≠ ε_2_ (i.e., neurons have different firing rates) and the coupling strength *g*_*syn*_ is not very strong.

We consider a minimal neuronal network to exhibit synchronized dynamics: two neurons mutually connected with excitatory synapses (Figure 2). These two neurons satisfy (Equations 1–5) and the synapses are described by Equation (6). We consider two weakly coupled neurons with a small difference in firing rate (that is, the frequency of oscillations of voltage) due to slightly different ε. Note that since ε_1_ ≠ ε_2_ and the coupling strength *g*_*syn*_ = 0.005 is weak, two cells are not fully synchronized. Thus, the synchrony is intermittent rather than perfect.

#### The effect of the peak value of activation time-constant

The magnitude of the voltage-dependent activation time-constant τ(*v*) of potassium current is inversely proportional to the parameter ε in such a way that the maximal value of τ(*v*) is 1/ε. We consider here how ε affects the durations of desynchronization events and accompanying changes in the average synchrony level and the mean frequency of spiking.

**Figure 3:**
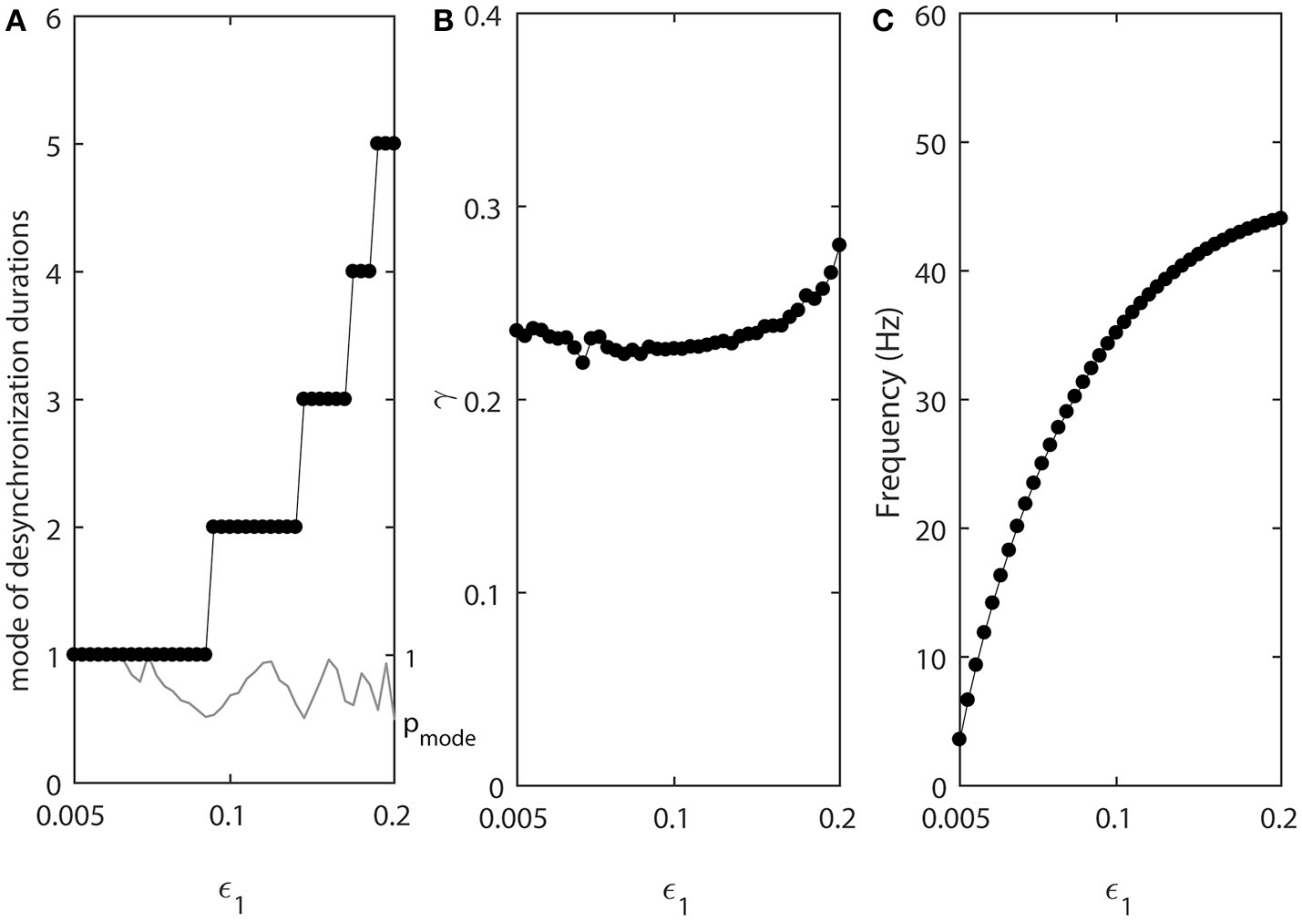
The effect of ε_1_ when ε_2_ = 1.2ε_1_. **(A)** Mode value of the durations of desynchronization events (black line with black dots) and the corresponding probability to observe the mode value (gray line). The high (close to one) value of probability at mode indicates that the desynchronizations of corresponding duration are strongly prevalent. **(B)** Synchronization strength index γ. **(C)** The mean frequency of activities of both neurons. Since ε_2_ = 1.2ε_1_, neuron 2 has slightly higher frequency than the mean frequency while the neuron 1 has slightly lower frequency than the mean frequency.

As the values of ε_1_ and ε_2_ increase, the fine temporal structure of synchronization changes as evident by the changes of the mode of the distribution of desynchronization durations (Figure 3A). Smaller values of ε promote short desynchronization intervals lasting for only one cycle of oscillations. On the contrary, the increase in ε leads to the increase of the mode of the distribution of desynchronization durations. That is, as ε increases, the most frequent desynchronization intervals are getting longer. Since the activation time-constant τ(*v*) is inversely proportional to ε, larger value of τ(*v*) (which promotes spike-like waveform in the model) promotes short desynchronization dynamics.

The synchrony strength γ experiences only very small variations (Figure 3B). This indicates that the durations of desynchronizations may be independent of the synchrony strength. The same level of synchrony may be reached with numerous short desynchronizations or few long desynchronizations.

The mean frequency (firing rate) grows substantially (Figure 3C). This is expected because the growth rate of *w* is proportional to ε (Equation 2). As a result, while desynchronization intervals measured in cycles of oscillations are longer for larger ε, their durations in the absolute time are not necessarily growing. We will address this issue below.

Note that the probability of the dominant duration of desynchronization events *p*_*mode*_ (thin gray line without dots in Figure 3A) is mostly close to 1 and always higher than 0.5. Thus, the desynchronization durations of the corresponding number of cycles is really dominant (because the sum of all probabilities of the durations of desynchronization events is 1).

#### The effect of the widths of voltage-dependence of the activation time-constant τ(*v*)

In the model, the parameter β is related to the width of the steady state activation function *w*_∞_(*v*) and range of activation constant τ(*v*), where τ(*v*) is substantially different from 0. The results presented below will show that it is mostly the widths of voltage-dependence of the activation time-constant τ(*v*) that matters for the properties of desynchronization durations. As β increases, the width of τ(*v*) increases. That is, the range of voltages, where the activation time-constant is different from 0, is getting larger. This may effectively bring τ closer to the maximal possible value (which is 1/ε in the model) for a larger range of voltage and thus for a longer time. Thus, similar to the decrease of ε, larger β will promote more “spiky” and less quasi-sinusoidal waveforms.

**Figure 4:**
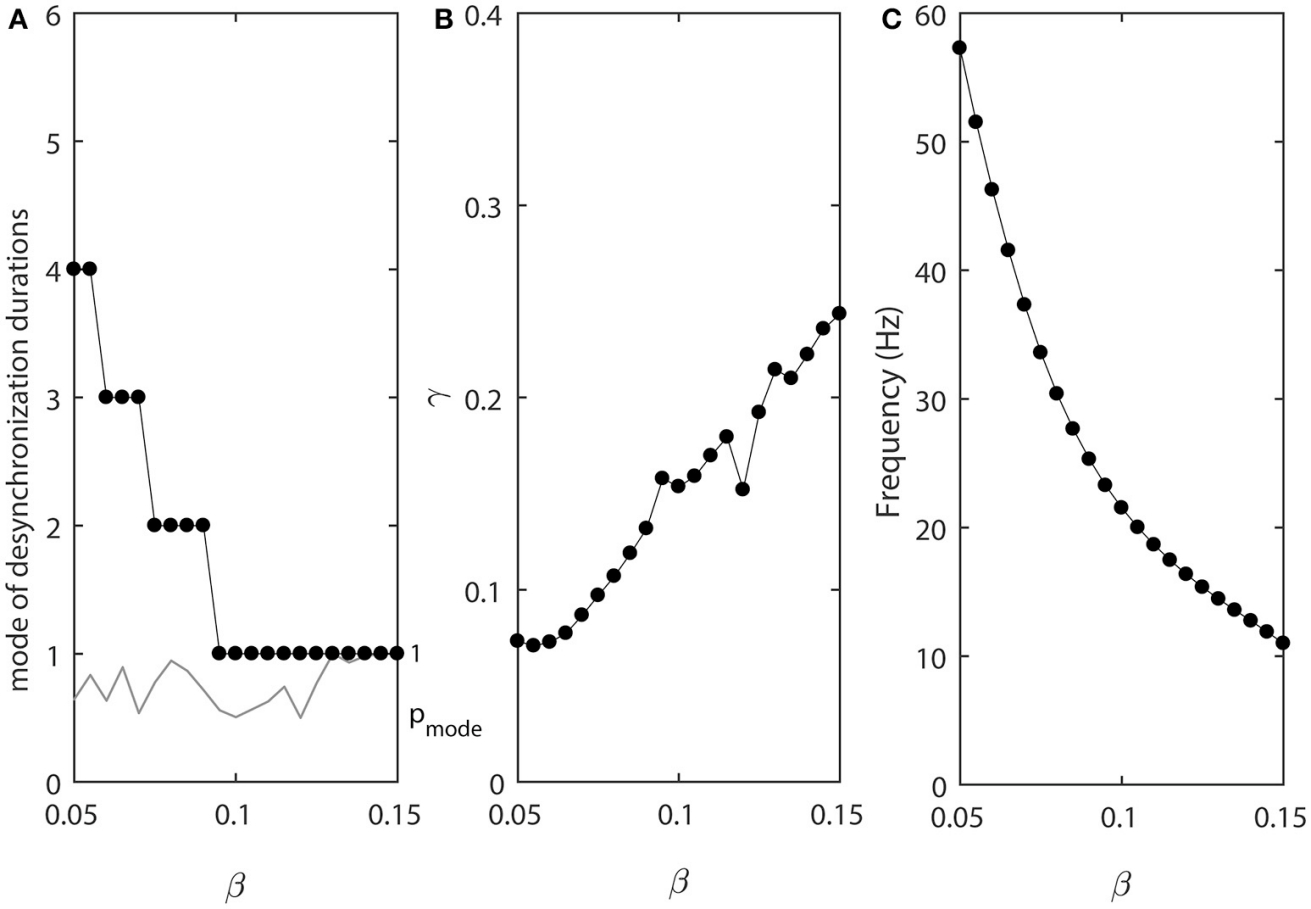
The effect of β when ε_1_ = ε and ε_2_ = 1.2ε. **(A)** Mode value of the durations of desynchronization events (black line with black dots) and the corresponding probability to observe the mode value (gray line). **(B)** Synchronization strength index γ. **(C)** The mean frequency of activities of both neurons.

Figure 4 shows how the parameter β affects the synchronized dynamics of coupled neurons. Larger values of β promote shorter desynchronization episodes (Figure 4A). There is also an effect on the synchrony strength (Figure 4B) and the frequency (Figure 4C). Shorter desynchronizations correspond to the higher synchrony level. As β is changing, the frequency is changing. This may mitigate the short desynchronization phenomena if desynchronization duration is measured in absolute units of time instead of cycles of oscillations. Nevertheless, similar to the case considered above, a change in parameter that leads to larger values of activation time-constant τ (increase in β) promotes desynchronizations of shorter durations [as signified by the high value of the probability at the mode of distribution of desynchronization durations (gray thin line in Figure 4A)].

#### The effect of the voltage of half-activation and maximal activation time-constant

We now consider the effect of *v*_*w*_1__ which is the midpoint of the steady state activation function *w*_∞_(*v*), that is the voltage at which half of the channels open. The same parameter defines the voltage at which the activation time-constant τ(*v*) peaks. Increase in *v*_*w*_1__ shifts both curves *w*_∞_(*v*) and τ(*v*) in the direction of higher voltages. The conductance will start to increase at higher voltage and at a later time, and will start to decrease at earlier time. Thus, larger value of *v*_*w*_1__ may be expected to have an effect analogous to decrease in τ.

**Figure 5:**
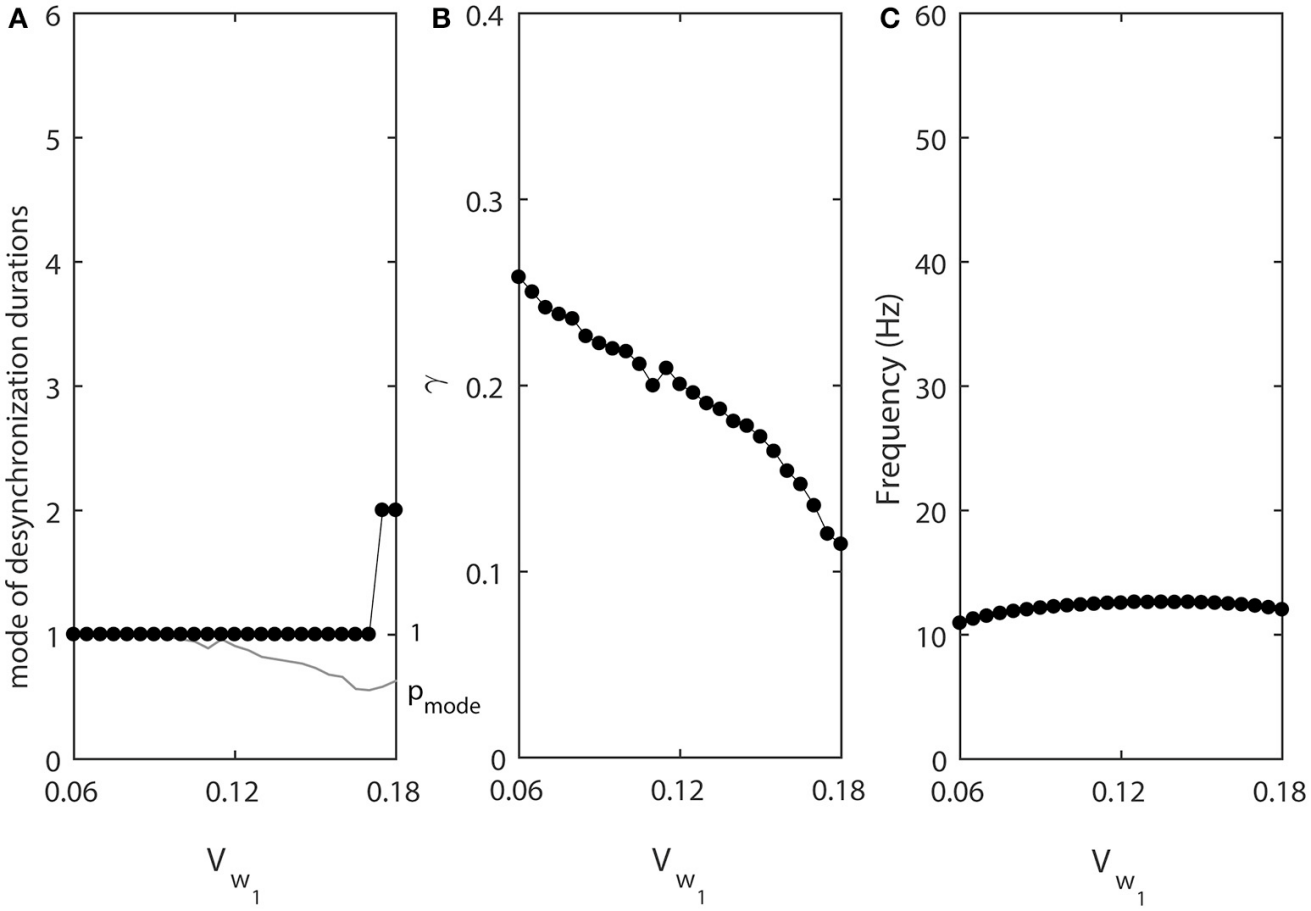
The effect of *v*_*w*_1__ when ε_1_ = ε and ε_2_ = 1.2ε. **(A)** Mode value of the durations of desynchronization events (black line with black dots) and the corresponding probability to observe the mode value (gray line). **(B)** Synchronization strength index γ. **(C)** The mean frequency of activities of both neurons.

The results of numerical simulation presented at the Figure 5 support this. Lower values of *v*_*w*_1__ promote shorter desynchronizations (Figure 5A). In this case, the synchronization strength is larger for short desynchronization dynamics (Figure 5B), but the frequency is almost constant (Figure 5C). Thus, the desynchronizations are short here not only if measured in the number of cycles (spikes), but also measured in absolute time units.

#### Changing desynchronization durations independently of both frequency and synchrony strength

The changes in desynchronization durations in the numerical experiments above are accompanied by changing either average synchrony strength or firing rate (or even both). Here we consider whether the desynchronization durations can vary independently of both synchrony strength and firing rate. To study this, we modify the Equations (4) and (5) so that the values of parameter β in equations for *w*_∞_(*v*) and τ(*v*) are not identical. This means the half-activation voltage is different from the voltage at which τ(*v*) has maximal value:

(9) $$\begin{array}{r} w_{\infty }\left(v\right)=\frac{1}{1+\text{exp}\left(-2\left(\frac{v-v_{w_{1}}}{\beta_{w}}\right)\right)}, \end{array}$$

(10) $$\begin{array}{r} \tau \left(v\right)=\frac{1}{\varepsilon }\frac{2}{\exp\left(\frac{v-v_{w_{1}}}{2\beta_{\tau }}\right)+\text{exp}\left(\frac{-\left(v-v_{w_{1}}\;\right)}{2\beta_{\tau }}\right)}. \end{array}$$

Smaller β_*w*_ makes the slope of the steady-state activation function *w*_∞_(*v*) larger, while smaller β_τ_ makes the width of the constant function τ(*v*) smaller. We let β_*w*_ and β_τ_ be changing in opposite directions. As β_*w*_ decreases from 0.134, β_τ_ increases from 0.061 at a different rate (β_*w*_ = 0.134 − 0.001*k* and β_τ_ = 0.061+0.0005*k*, where *k* = 0, 1, …, 40.). For other parameters, we use ε_2_ = 1.3ε_1_, ε_1_ = 0.03, *I*_*app*_ = 0.04, *g*_*syn*_ = 0.005, *v*_*w*_1__ = 0.07. These changes of β_*w*_ and β_τ_ are not necessarily biologically realistic, but they allow us to explore whether the changes of desynchronization durations must covary with the changes of average synchrony or firing rate.

**Figure 6:**
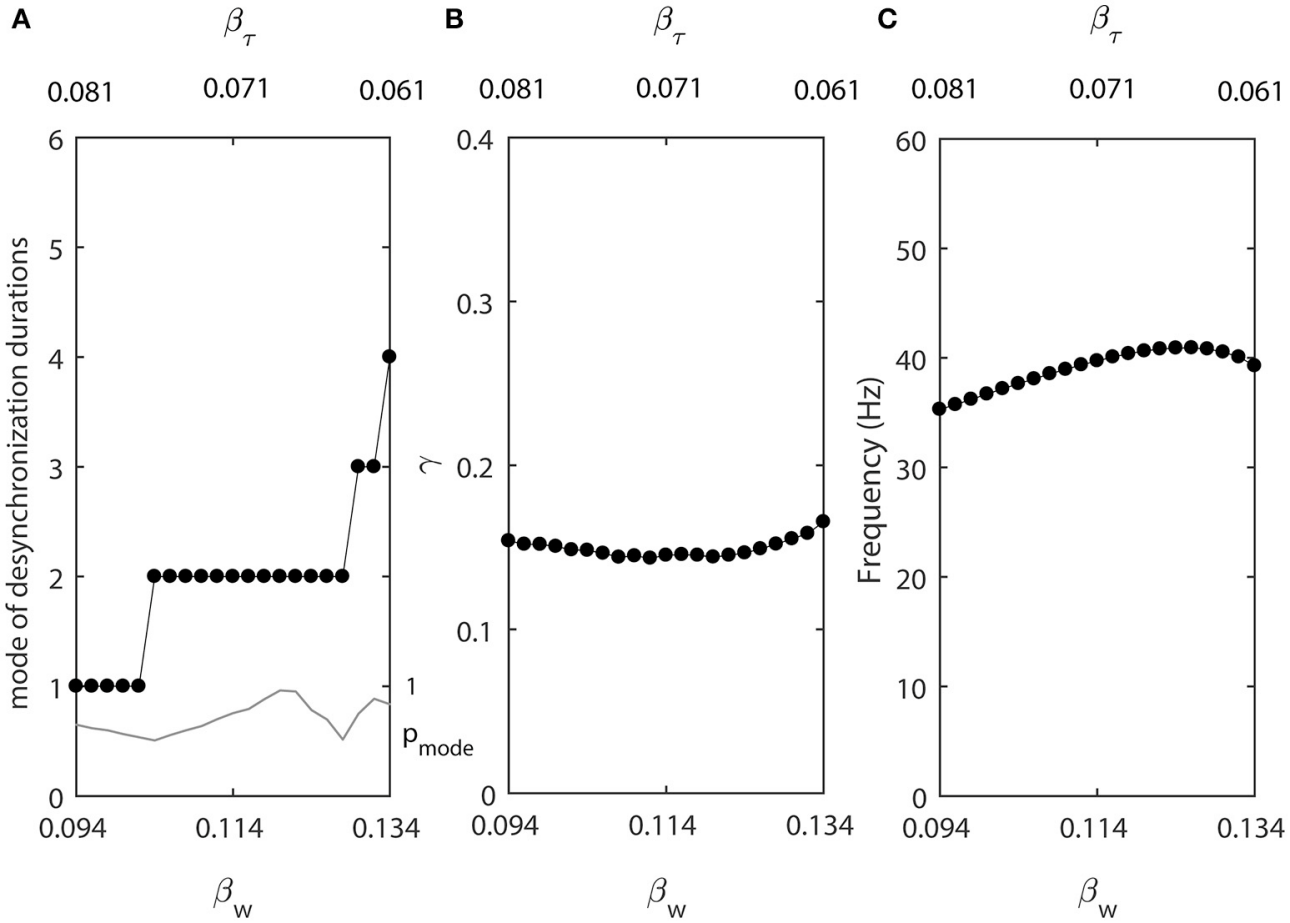
Changing synchronization durations independently from synchrony strength and firing rate. **(A)** Mode value of the durations of desynchronization events (black line with black dots) and the corresponding probability to observe the mode value (gray line). **(B)** Synchronization strength index γ. **(C)** The mean frequency of activities of both neurons.

Figure 6 shows that in this case the synchrony strength and the firing rate are almost constant while the mode of desynchronization durations changes drastically. In other words, simultaneous variations of the width of *w*_∞_(*v*) and τ(*v*) vary the distribution of desynchronizations independently from synchrony strength and firing rate. Thus, the same level of synchrony strength may be supported either with many short desynchronizations or few long desynchronizations regardless of whether the durations of desynchronizations are measured in cycles of oscillations or in absolute time units.

### Short desynchronization dynamics and synchronization threshold

**Figure 7:**
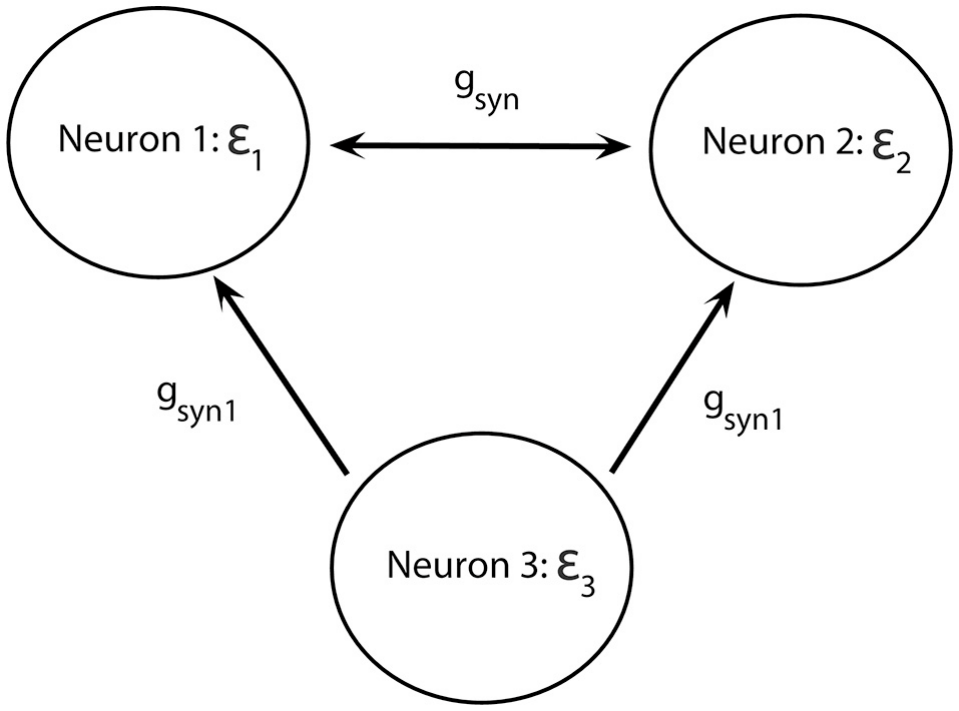
Diagram of a minimal network of excitatory coupled neurons receiving common synaptic input. Neurons 1 and 2 have different firing rates. They are mutually coupled through excitatory synapses and receive synaptic input from neuron 3.

To study potential functional advantages of short desynchronizations dynamics, we will consider two mutually excitatory connected neurons (as before) receiving common synaptic input from a third neuron: neuron 3 excites neurons 1 and 2 through the excitatory synapses (but does not get any feedback, Figure 7).

We consider two different versions of three-neuron networks in Figure 7. In the first version, the parameters are selected in such a way that when *g*_*syn*1_ = 0 neurons 1 and 2 exhibit dynamics with mostly short desynchronizations. The second version exhibits partially synchronized dynamics with the most common desynchronization intervals lasting for 4 cycles of oscillations when *g*_*syn*1_ = 0. In other words, we consider how two coupled neurons exhibiting either short desynchronization dynamics or longer desynchronization dynamics respond to the common synaptic input.

One network has β_*w*_ = 0.094 and β_τ_ = 0.081, this is the left end of the horizontal axis in Figure 6A. The mode of desynchronization durations is just 1 and we will call this network “cycle 1” network (short desynchronizations network). The other network has β_*w*_ = 0.134 and β_τ_ = 0.061, this is the right end of the horizontal axis in Figure 6A. The mode of desynchronization durations is 4 and we will call this network “cycle 4” network (longer desynchronizations network). It is important to note that both networks have almost the same synchrony strength (Figure 6B) and firing rate (Figure 6C). So, except the difference in desynchronization durations, the dynamics of two networks are similar. That is, they have the same synchrony level and the same period of oscillations in the absence of synaptic input from the neuron 3.

In the numerical experiments, ε_1_ = 0.03 and ε_2_ = 1.3ε_1_, the same values as used in Figure 6. We consider four different values of the firing rate in the neuron 3: $\varepsilon_{3}=\left\{0.5\varepsilon_{1},\;\frac{\varepsilon_{1}+\varepsilon_{2}}{2},\;1.5\varepsilon_{1},\;2\varepsilon_{1}\right\}$. So, the firing rate in neuron 3 is either substantially lower than in neurons 1 and 2, equals to the average of firing rates of neurons 1 and 2, or is higher than firing rates in neurons 1 and 2. All other parameters of neurons 1, 2, and 3 are the same and fixed as those used in Figure 6.

Now let us consider these two networks as the common input to neurons 1 and 2 is getting stronger due to increase of *g*_*syn*1_ from zero (while *g*_*syn*_ = 0.005 is fixed, that is, the coupling between neuron 1 and neuron 2 is relatively weak). As synaptic input from neuron 3 to neurons 1 and 2 is getting stronger, neurons 1 and 2 are becoming more synchronous and will eventually be in full synchrony with each other due to common synaptic input and mutual synaptic coupling.

**Figure 8:**
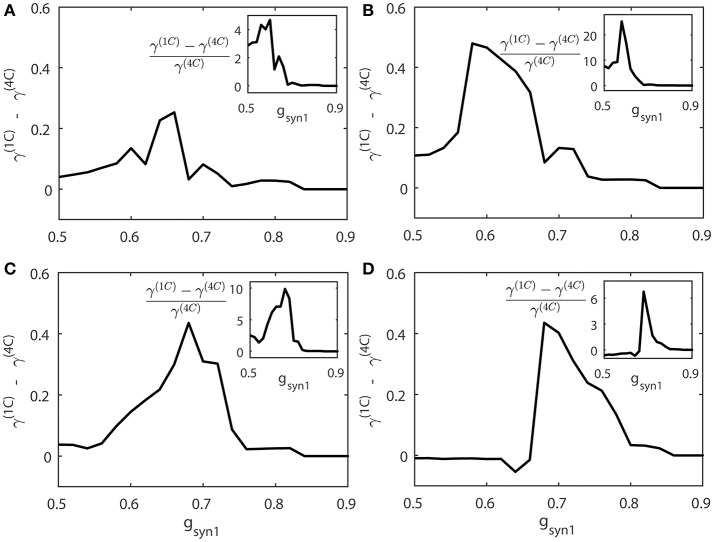
The synchrony difference γ^(1*C*)^ − γ^(4*C*)^ is plotted for different strength of synaptic input *g*_*syn*1_, normalized synchrony difference ($\frac{\;\gamma^{\left(1C\right)}-\;\gamma^{\left(4C\right)}}{\;\gamma^{\left(4C\right)}}$) is presented at the inserts (γ^(1*C*)^ and γ^(4*C*)^ represent the synchrony index γ for “cycle 1” (short desynchronizations) and “cycle 4” (long desynchronizations) networks, respectively). Subplots **(A–D)** are for different values of the firing rate of incoming signal, corresponding to $\varepsilon_{3}=\left\{0.5\varepsilon_{1},\;\frac{\varepsilon_{1}+\varepsilon_{2}}{2},\;1.5\varepsilon_{1},\;2\varepsilon_{1}\right\}$ respectively.

We compute the synchrony index γ for “cycle 1” and “cycle 4” networks (γ^(1*C*)^ and γ^(4*C*)^ respectively) when increasing values of *g*_*syn*1_. To study how differently these networks are synchronized, we consider the absolute and relative difference of synchronization indices γ^(1*C*)^ and γ^(4*C*)^ for different values of *g*_*syn*1_. Figure 8 presents the averages of γ^(1*C*)^ − γ^(4*C*)^ (thick solid line) and $\frac{\;\gamma^{\left(1C\right)}-\;\gamma^{\left(4C\right)}}{\;\gamma^{\left(4C\right)}}$ (the insert figure). Both quantities indicate how much synchronization in “cycle 1” (short desynchronizations) network is stronger than synchronization in “cycle 4” network when they receive the same synaptic input from the neuron 3.

When this input is weak (*g*_*syn*1_ is small), γ^(1*C*)^ and γ^(4*C*)^ are close to each other. When *g*_*syn*1_ is large, γ^(1*C*)^ and γ^(4*C*)^ are again close to each other because both networks are necessarily strongly synchronous due to strong input. However, for the values of *g*_*syn*1_ between zero and synchronization threshold value, γ^(1*C*)^ − γ^(4*C*)^ is large and positive. So the networks exhibiting short desynchronizations dynamics in the absence of input (“cycle 1” networks) reach either the same synchrony levels or higher synchrony levels than long desynchronization (“cycle 4”) networks for the same strength of synaptic input *g*_*syn*1_. This phenomenon is observed regardless of the firing rate in presynaptic neuron 3 (i.e., regardless of ε_3_). Sometimes this difference in the synchronization strength is moderate, but sometimes it is quite substantial (see Figure 8). The synchrony index is bounded by one from above, so the magnitude of the phenomenon is more emphasized by observing the relative value of synchronization index difference (inserts in Figure 8).

**Figure 9:**
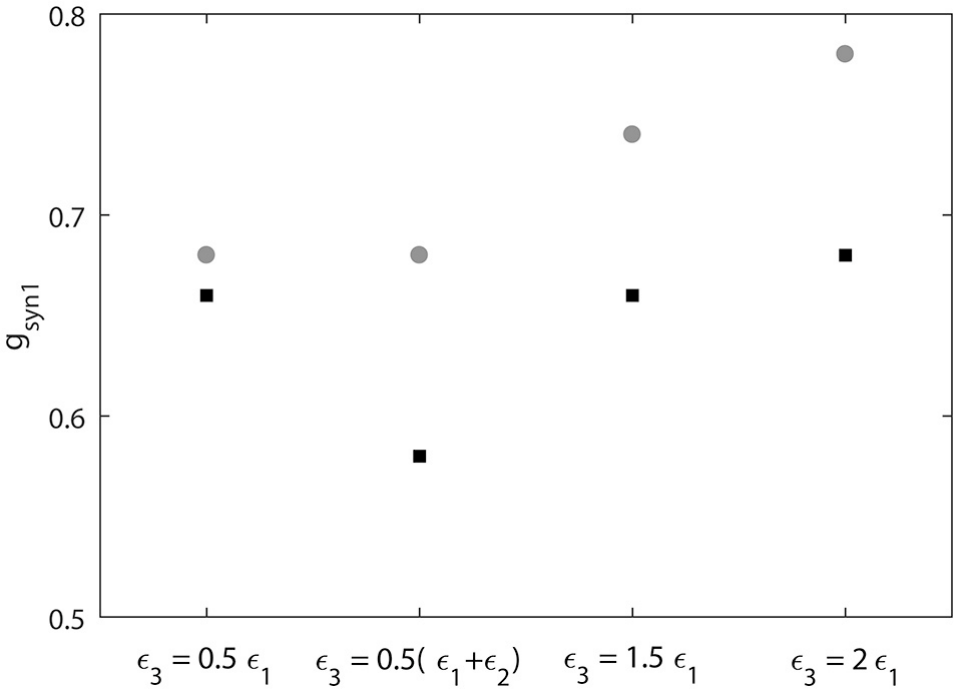
Threshold value of synaptic strength *g*_*syn*1_ to reach synchornized dynamics without desynchronization events for different values of ε_3_. Black squares represent the critical value of *g*_*syn*1_ for short desynchronization (“cycle 1”) network and the gray circles represent the critical value of *g*_*syn*1_ for long desynchronization (“cycle 4”) network.

We also measure the threshold value *g*_*syn*1_ for two neruons to reach synchornized dynamics without desynchronization events (Figure 9). This does not imply complete synchrony, but implies only small deviations between the phases of two signals, so that it is small enough to have no desynchonization events. As can be seen in Figure 9, the computed synchrony thresholds for short desynchronization (“cycle 1”) network were lower than the synchrony thresholds for long desynchronization (“cycle 4”) network for all considered firing rates (all possible ε_3_).

The results presented in Figures 8, 9 indicate that with average synchrony level and mean firing rate being equal, neural systems with short desynchronization dynamics reach higher synchrony for the same synaptic input strength and need weaker inputs to be synchronized than neural systems with long desynchronization events.

## Discussion

### Cellular mechanisms of short desynchronization dynamics

Imperfect synchrony is widely observed in the activity of neural networks of the brain. New time-series analysis techniques showed that intervals of synchronous dynamics are interspersed between desynchronized episodes, and most desynchronized episodes are very short (see references in Introduction). This stereotyped fine temporal structure of neural synchronization is not an artifact of the analysis method because other types of patterning of synchronization are possible in non-neural coupled oscillators (Ahn et al., 2011; Rubchinsky et al., 2014).

The present study provides potential mechanisms for this type of temporal patterning of neural synchrony. We varied several parameters of potassium conductance and identified conditions leading to the intermittent neural synchrony with predominantly short desynchronization episodes similar to experimental ones. All these conditions (large peak value of activation time-constant, large width of dependence of activation time-constant on voltage, lower values of voltage for peak activation time-constant) lead to the relatively large values of the activation time-constant τ(*v*) in the right range of voltages. The large value of τ(*v*) leads to the delay in activation of potassium current, so that a sharp spike can be generated. And, as our results show, it promotes the short desynchronizations dynamics.

The results of the computational modeling also indicate that the distribution of desynchronization durations may be independent of the synchronization strength. The same synchrony strength may be achieved with desynchronizations of different durations. Moreover, our results regarding comparison of synchronization in networks exhibiting short desynchronizations and long desynchronizations are obtained for the case when not only average synchrony level is the same, but the period of oscillations (the firing rate) is the same. By appropriate adjustment of model parameters we dissociated the effects of frequency of oscillations and of average synchrony strength from the effects of fine temporal patterning of synchronized dynamics.

These model-based observations fit with experimental observations of the changes in the distribution of desynchronization durations in prefrontal cortex-hippocampal synchrony in behavioral sensitizations experiments (Ahn et al., 2014). In these experiments, the desynchronization durations were predominantly short and their distributions were altered after psychostimulant administration, while the average synchrony levels stayed the same.

The “spikiness” of oscillations of transmembrane voltage is a very generic property of many neurons, which relies on the fast activation of current with high reversal potential and slow activation of current with low reversal potential. Our results show that the same conditions that promote short desynchronization dynamics promote the characteristically sharp shape of an action potential. This may explain why very different neural systems exhibit short desynchronization dynamics, as we described in Introduction.

### Limitations of the modeling approach

We use a very simple model of a neuron and very simple model of a network. There are many factors, which may affect synchronous dynamics of neural activity, yet they are not represented in the model. Other important factors, which affect neural synchrony, are different membrane currents and their properties (we have a model with just two conductances and consider only several parameters of one conductance) and the size of the network (we have a very small network). Heterogeneity of the networks is also important (we have a very minimal representation of heterogeneity). Synaptic plasticity is not incorporated in our model (and is the subject of the future research). Finally, noise may affect temporal patterns of synchrony, which is not considered in this study either.

However, even though these factors are not incorporated in the model (which captures only some very basic mechanisms of neural activity), the model is able to generate realistic synchrony patterns. So, the right way to interpret the modeling results is to see what these basic mechanisms are capable of. These modeling results suggest that these very basic neural mechanisms are capable of explaining the properties of experimentally observed intermittency of neural synchrony. As we discussed above, short desynchronization dynamics has been observed in several different neural systems. An ability of a minimal neural network considered here to describe the properties of the intermittent synchrony (which is common to all those systems) is probably an indicator that the general neural mechanisms built in the model are adequate to the considered phenomena.

Inhibition is playing an important role in neural synchronization, but is not considered in our model. The experimental data discussed here were collected from cortical and subcortical networks with excitatory and inhibitory synapses. It will be interesting to see how the intermittent patterns of synchrony are affected by inhibitory synapses.

We would also like to note that our earlier study with more advanced neural and network model (which included excitatory and inhibitory synapses) did provided a quantitatively adequate description of the short desynchronization dynamics at the beta-band oscillations in the basal ganglia in Parkinson's disease (Park et al., 2011). The present modeling study is not designed to provide a quantitative description of a specific experiment, but rather it provides a qualitative description of common aspects of neural synchrony in different neural systems.

### Potential functional significance of short desynchronization dynamics

Our computational results suggest one way of how short desynchronization dynamics can be beneficial for neural systems. With two important properties of dynamic (average synchrony strength and firing rate) being equal, neural systems with short desynchronizations are easier to synchronize with common synaptic input. We showed that the same strength of common synaptic input leads to larger synchrony level in short desynchronization system. In other words, short desynchronization dynamics allows reaching a pre-set synchrony level with weaker input. So, if a strong synchrony is needed, systems with short desynchronizations will reach the pre-set synchrony strength with weaker inputs compared to longer desynchronizations.

Given the functional importance of synchronization in many neural systems (see references in Introduction), short desynchronization dynamics may allow for efficient regulation of synchrony levels. While the same level of synchrony may potentially be achieved with few long desynchronization episodes as well as with many short desynchronization episodes, only short desynchronization dynamics is experimentally observed in the neural synchrony in the brains. Our modeling results suggest that this short desynchronizations dynamics is easier to control with synaptic input. Thus, very basic properties of delayed rectifier potassium current (its delayed activation) is likely to promote efficient formation and break-up of synchronized assemblies.

## Author contributions

LR conceived research; SA and LR designed research; SA performed numerical simulations, SA and LR analyzed and interpreted the results; SA and LR wrote the manuscript.

### Conflict of interest statement

The authors declare that the research was conducted in the absence of any commercial or financial relationships that could be construed as a potential conflict of interest.
